# The investigation of constraints in implementing robust AI colorectal polyp detection for sustainable healthcare system

**DOI:** 10.1371/journal.pone.0288376

**Published:** 2023-07-12

**Authors:** Haitao Bian, Min Jiang, Jingjing Qian

**Affiliations:** 1 College of Safety Science and Engineering, Nanjing Tech University, Nanjing, Jiangsu, China; 2 KLA Corporation, Milpitas, California, United States of America; 3 Department of Gastroenterology, The Second Hospital of Nanjing, Nanjing University of Chinese Medicine, Nanjing, Jiangsu, China; Ibn Zohr University: Universite Ibn Zohr, MOROCCO

## Abstract

Colorectal cancer (CRC) is one of the significant threats to public health and the sustainable healthcare system during urbanization. As the primary method of screening, colonoscopy can effectively detect polyps before they evolve into cancerous growths. However, the current visual inspection by endoscopists is insufficient in providing consistently reliable polyp detection for colonoscopy videos and images in CRC screening. Artificial Intelligent (AI) based object detection is considered as a potent solution to overcome visual inspection limitations and mitigate human errors in colonoscopy. This study implemented a YOLOv5 object detection model to investigate the performance of mainstream one-stage approaches in colorectal polyp detection. Meanwhile, a variety of training datasets and model structure configurations are employed to identify the determinative factors in practical applications. The designed experiments show that the model yields acceptable results assisted by transfer learning, and highlight that the primary constraint in implementing deep learning polyp detection comes from the scarcity of training data. The model performance was improved by 15.6% in terms of average precision (AP) when the original training dataset was expanded. Furthermore, the experimental results were analysed from a clinical perspective to identify potential causes of false positives. Besides, the quality management framework is proposed for future dataset preparation and model development in AI-driven polyp detection tasks for smart healthcare solutions.

## 1. Introduction

Colorectal cancer (CRC) is believed to be strongly associated with urban lifestyle and socioeconomic development [1]. Established risk factors for CRC initiation and progression include low physical activity, overweight and obesity, and dietary habits, all of which are linked to lifestyle changes during urbanization [2]. Moreover, studies have also suggested an association between variations in specific gene polymorphisms and the incidence of colorectal cancer [3, 4]. Notably, CRC is now the third most prevalent cancer worldwide and has risen to become the second leading cause of cancer-related death [5, 6]. The steady increase in CRC incidence could place a substantial public health burden on society and challenge the development of a sustainable healthcare system, as considerable medical resources would be required for cancer treatment [7, 8].

Colorectal polyps are swellings originating from the mucosal epithelium of the intestine and can be classified as inflammatory polyps, hyperplastic polyps (HPP), or colorectal adenomas (CRA) from a clinical perspective. Approximately 85% of CRCs develop from CRA through the adenoma-carcinoma sequence over 5–15 years [9]. The primary screening methods for colorectal cancer (CRC) currently encompass colonoscopy, wireless capsule endoscopy, and fecal occult blood tests (FOBT). Colonoscopy, which allows for the visual observation of lesions, is considered the gold standard for CRC screening and surveillance among these techniques. Consequently, enhancing the adenoma detection rate (ADR) and polyp detection rate (PDR) in colonoscopy is crucial for CRC prevention [10]. If polyps are detected during colonoscopy, endoscopic polypectomy or endoscopic mucosal resection (EMR) can serve as effective interventions to reduce the risk of cancerous lesions. Furthermore, a suitable therapeutic or follow-up plan would be formulated based on the pathology [11]. However, studies have reported that up to 30% of adenomas are missed during conventional colonoscopy [12]. Factors such as poor bowel preparation, image quality, and polyp appearance, location, or size all limit the effectiveness of visual inspection during colonoscopy. Additionally, endoscopists’ training, experience, fatigue, and subjectivity may influence the interpretation of colonoscopy images. Unfortunately, if polyps are ignored during the initial colonoscopy, there is a high risk that the polyp may become cancerous while awaiting the next scheduled examination.

For decades, computer-aided diagnosis (CAD) has been considered a supplementary technique in medical image processing [13]. In the initial stages of these investigations, traditional feature extraction and machine learning algorithms from computer vision were applied to develop automated recognition programs for nodules, polyps, tumors, and other lesions [14]. For example, Wang et al. proposed a support vector machine (SVM) based lung nodules detection method for computed tomographic scans [15]. An extreme learning machine (ELM) classifier was introduced for detecting breast tumors in digital mammography using extracted textural and morphological features [16]. Although the performance of these detection models was acceptable on test datasets, their reliability in practical applications remained uncertain. Manual feature extraction necessitated collaboration between computer graphics scientists and medical experts, often resulting in the learning of only shallow image features. To accurately characterize lesion images, it is essential to observe a substantial number of samples since lesion characteristics vary significantly among patients. Besides, polyps are complex in size, appearance, and texture, and certain objects in the colorectal environment may be mistaken for polyps. Therefore, designing a reliable and accurate machine learning-based clinical diagnosis program for polyp detection is challenging without sufficient high-quality colonoscopy images and expertise in feature engineering.

Convolution neural networks (CNN) and other deep learning architectures are promising in computer vision tasks, such as classification, object detection, and image segmentation. The region-based CNNs, including R-CNN, Fast R-CNN, Faster-RCNN, and Mask R-CNN, exhibit exceptional performance in object localization and recognition [17–20]. As the representative one-stage object detection approaches, the YOLO and SSD can achieve real-time detection with a compromise of prediction accuracy [21, 22]. EfficientDet incorporates compound scaling, bidirectional feature pyramid network (BiFPN), and weighted feature fusion to balance the accuracy and computational efficiency in object detection [23]. The Swin Transformer also demonstrated the architecture’s exceptional performance in various computer vision tasks [24]. One-stage object detection models eliminate the need for a region proposal network, directly outputting category labels and location information as bounding boxes (*x*_*i*_, *y*_*i*_, *w*_*i*_, *h*_*i*_) for the input image. Although early YOLO models offer rapid target detection, they struggle to learn fine features compared to region-based R-CNN series networks. Through rigorous experimentation and refinement, YOLOv4 has evolved to possess a comprehensive structure consisting of a backbone, neck, and head. Consequently, the state-of-the-art YOLO models closely rival their two-stage competitors in accurately classifying and locating targets while preserving its speed advantage. Moreover, the performance of YOLOv5 can be enhanced by incorporating (BiFPN) to augment feature fusion and refine the model’s capabilities in a recent study [25].

The ground-breaking advancements in object detection through deep learning have raised significant interest in incorporating CNN into colorectal polyp detection. Rahim et al. developed a deep CNN model for colorectal polyps detection [26]. Chen et al. created a self-attention-based Faster R-CNN framework for detecting polyps in colonoscopy images [27]. Pacal and Karaboga proposed a deep learning polyp detection model adapted from the YOLO architecture [28]. Meanwhile, a YOLOv3 based polyp detection algorithm is presented, utilizing a transfer learning strategy with negative samples to enhance the model performance [29]. Moreover, Thomaz et al. introduced data augmentation techniques to expand the limited polyp image dataset and improve polyp detection performance using deep CNNs [30]. Ribeiro et al. employed transfer learning for polyp detection to evaluate the prevailing CNN models across multiple public datasets [31]. Furthermore, object detection algorithms have been applied in automated prostate cancer grading and diagnosis systems [32]. Previous studies have shown the outstanding polyp detection capabilities of CNN models on both public and private datasets from a model optimization standpoint. However, it does not necessarily align with the clinical application requirements.

It is widely acknowledged that deep learning requires a substantial volume of training data to build a robust and reliable model. Acquiring adequate and high-quality datasets in medical image processing tasks must take into account the protection of patient privacy and the need to obtain approval from the ethics review board [33]. Furthermore, the annotation process demands expertise from medical professionals as well as a well-structured management framework to ensure quality. Although research on deep neural networks for medical image analysis continues to grow, uncertainties remain regarding the clinical applicability of these algorithms. Specifically, the health care community is uncertain about the diversity, comprehensiveness, and transparency of the polyp samples in the training dataset, the criteria for dataset annotation, and the dependability of the developed deep learning models.

This study implements the YOLOv5 object detection model to develop a polyp detection application using publicly accessible colonoscopy datasets. The model performance is assessed using standard computer vision metrics. The effects of varying depth and width configurations of the network layers are evaluated in subsequent experiments. The original training dataset is extended to evaluate the significance of training data diversity for contemporary polyp detection models development. The experimental results are analyzed from a clinical perspective to bridge the gap between computer vision and the medical community in AI assisted polyp detection.

The rest of this paper is organized as follows. Section 2 introduced the methodology of developing a CNN-based polyp detection model and the primary components of the proposed YOLOv5. Section 3 describes the experiments to assess performance from both model optimization and clinical perspectives, including the presentation of experimental results and analysis. Conclusions from this research are drawn in Section 4.

## 2. Materials and methods

### 2.1 CNN based polyp detection

**Fig 1:**
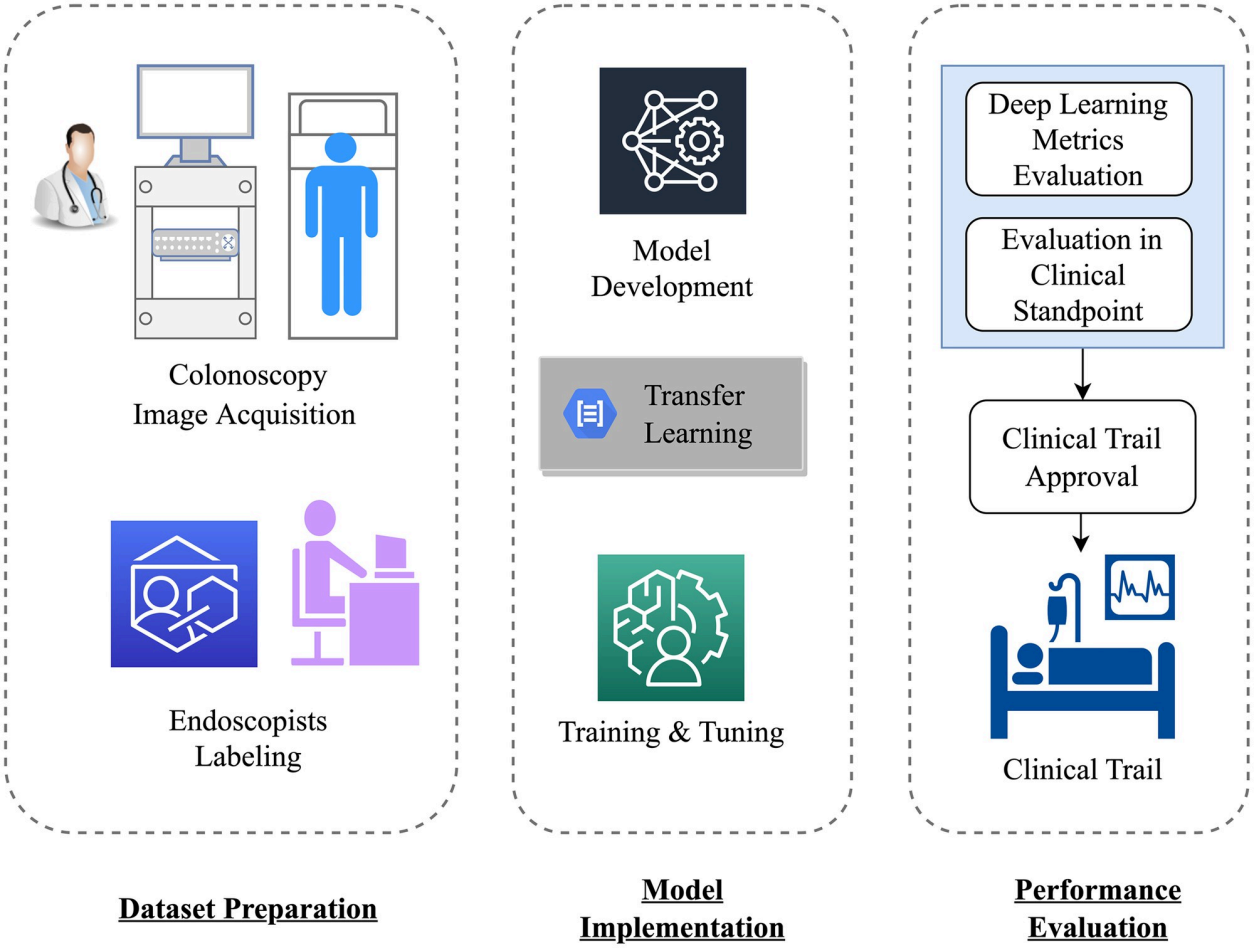
Overall schematic of deep learning based polyp detection.

Colonoscopy image acquisition and selection constitute the foundation for developing deep learning models for polyp detection. There are several common consensuses for preparing datasets, for instance, only patients without a history of colorectal surgery, gastrointestinal tumors, inflammatory bowel disease, and contraindications for gastrointestinal endoscopy are eligible to participate in the program. Polyp images should be captured at various locations within the bowel, such as the left colon, right colon, and rectum. A sufficient number of positive and negative samples, with appropriate distribution, is necessary for constructing a high-quality training dataset. Moreover, endoscopists need clear guidelines or standards for dataset preparation and polyp labelling in the images. The annotation process must include the ground truth for all polyps present in each image. In this manner, the input data quality for the target detection model could be ensured. Fig 1 presents an overview of the proposed deep learning polyp detection model development.

The polyp detection model aims to facilitate clinical diagnosis in colonoscopy videos, necessitating a balance between accuracy and speed in the proposed model. Upon establishing a suitable detection framework, the acquired dataset is partitioned into training, validation, and test sets, enabling the optimization of model structure and parameters. Due to the limited sample size in the polyp dataset, pre-trained weights from the Microsoft COCO dataset are employed to initialize the parameters for training the polyp detection model.

Evaluating the model’s detection performance requires more than merely examining precision, recall, F-score, and other general object detection metrics. Instead, endoscopists must assess the results to determine whether the proposed polyp detection method satisfies the practical requirements of clinical applications. The aforementioned research plan is designed to examine the implemented model, with a discussion of opportunities and challenges associated with deep CNN polyp detection from a clinical perspective.

### 2.2 Dataset preparation

The quality and variety of datasets play a crucial role in training deep CNN-based polyp detection models. Fortunately, ongoing efforts from academic, medical, and other communities have led to the creation of numerous public polyp datasets, such as CVC-ColonDB [34], ETIS-Larib [35], CVC-ClinicDB [36], SUN Database [37], ASU-Mayo Database [38], Kvasir-SEG [39], PICCOLO [40]. The growth of these datasets has considerably accelerated the progress of deep learning algorithms for polyp detection, with numerous studies relying on them for their investigations. Concurrently, researchers are also exploring polyp detection algorithms using private datasets acquired from colonoscopies at their own or affiliated hospitals. This paper employs a selection of the accessible public datasets to execute the proposed research plan, and the details of the datasets are shown in Table 1.

**Table 1:** The details of the selected datasets in polyp detection.

Dataset	Content	Original Mark	Resolution	Release
**CVC-ColonDB** [34]	300 images	Binary Mask	574 × 500	2012
**GIANA2017** [41]	23 videos	Binary Mask	Variable	2017
**GLRC** [42]	41 videos	Binary Mask	768 × 576	2016
**KUMC** [43]	76 videos	Bounding Box	592 × 464	2021
**Kvasir-SEG** [39]	1000 images	Binary Mask	Variable	2020
**CP-CHILD-A** [44]	8000 images	None	256 × 256	2020

In most instances, colonoscopy and chromoendoscopy do not provide conclusive evidence for characterizing polyps in preparation for subsequent procedures. Pathological examination remains necessary for determining the nature of the lesions. This study primarily investigates the presence or absence of polyps in the dataset, while further classification and segmentation tasks are not addressed at this stage. The GIANA2017 Datasets, KUMC, CVC-ColonDB, GLRC are preprocessed and delineated by Wang and colleagues, containing 37899 images and 35754 labeled files [45]. The Kvasir-SEG dataset contains1000 images with annotations. The CP-CHILD-A dataset specifically gathers colonoscopy polyp images from pediatric patients without annotation provided. In this study, the polyps present in the selected CP-CHILD-A images are labeled in the data preparation process.

### 2.3 Object detection model

#### 2.3.1 Brief model structure

**Fig 2:**
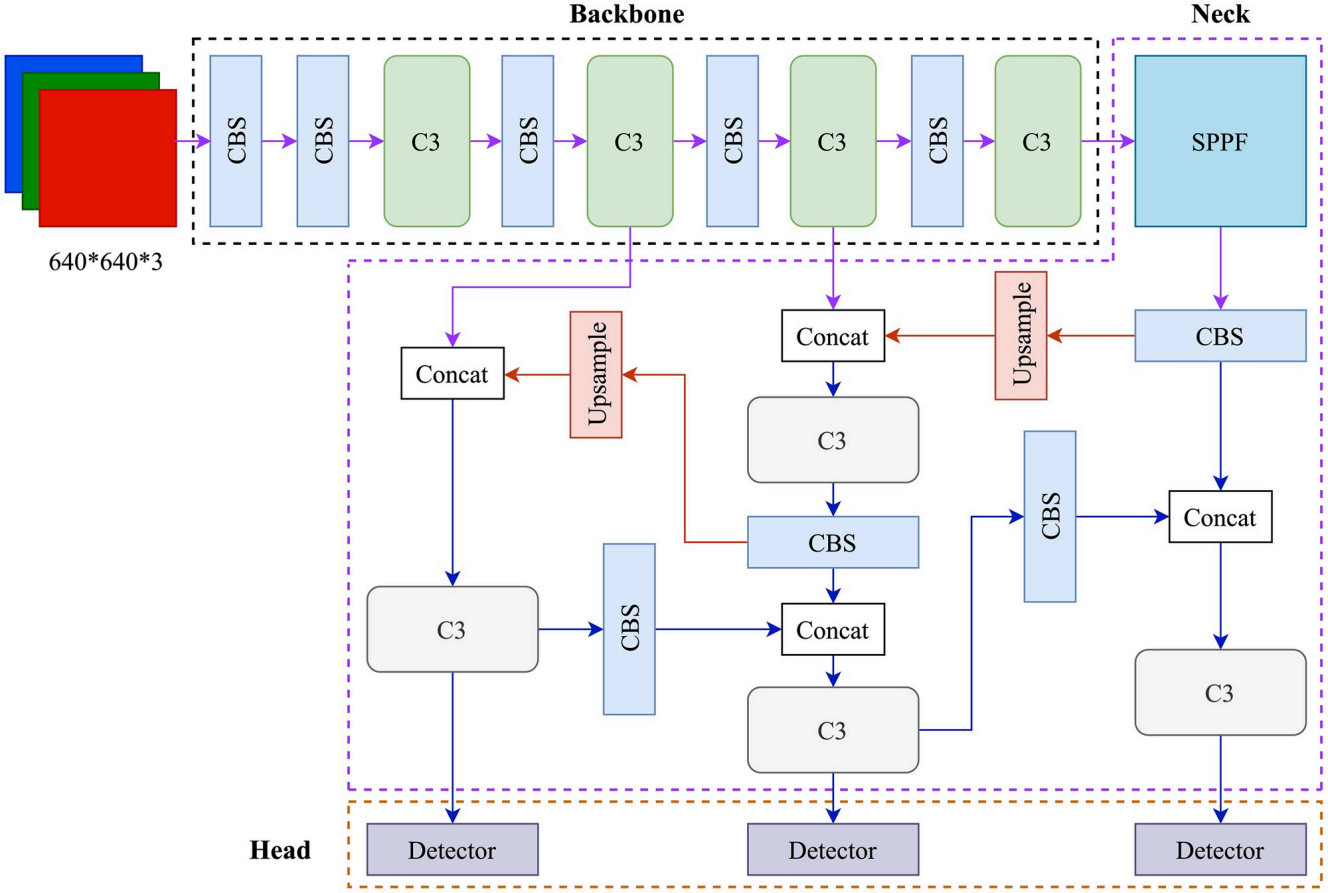
The brief structure of implemented YOLOv5 object detection model.

The YOLOv5 represents the state-of-the-art in proposal-free object detection models. In comparison to its early versions, YOLOv5 enhanced the detection accuracy and speed through the incorporation of the latest computer vision and deep learning technologies [46]. The YOLOv5 consists of three primary components: backbone, neck, and head. The backbone extracts feature representations from images, and is designed to minimize computational cost without compromising detection accuracy. The neck generates feature pyramids, enabling the model to capture objects of varying sizes and scales. The head is responsible for making predictions by applying anchor boxes to feature maps, yielding the final output. The YOLOv5 brief structure is illustrated in Fig 2.

The YOLOv5 backbone employs four C3 modules, functioning as a feature extraction network that generates feature maps from input images. The C3 module is derived from the Cross-Stage Partial Network (CSPNet) architecture, incorporating three standard convolutional layers and multiple bottleneck blocks [47]. Generally, CNNs are expected to perform better in computer vision tasks with increased width and depth of network layers. The CSPNet partitions the shallow feature map of the base layer into two sections in the channel dimension, one is propagated backward through the feature extraction module, and the other is directly merged with the module’s output. CSPNet’s aim is to create a lightweight CNN that enables richer gradient combinations, thereby enhancing learning capabilities. Image classification accuracy is improved, and computation is reduced when applying the CSPNet architecture to ResNet [48], DenseNet [49], and other CNNs. The CBS blocks comprise three modules, including convolution (Conv2d), batch normalization, and Sigmoid Linear Unit (SiLU) activation function:

$$\text{silu}\left(\text{x}\right)=\text{x}\;*\;\sigma \left(\text{x}\right)$$ (1)

where σ(x) is the logistic sigmoid.

The neck consists of a Spatial Pyramid Pooling Fast (SPPF) module and a modified Path Aggregation Network (C3-PANet). It concatenates feature maps from various layers of the backbone network before forwarding them to the head. The SPP layer utilizes multiple sliding windows to max pooling the feature maps of the upper convolutional layer from different sizes, and get several separate results to aggregate a fixed-length output [50]. Spatial Pyramid Pooling (SPP) is designed to handle images with varying scales, sizes, and aspect ratios, while features extracted at multiple scales enhance the deep neural network’s robustness and accuracy in image classification and object detection. In previous YOLO models, the SPP expands the receptive field of the backbone features, ensuring accurate target detection at various input scales. For YOLOv5, the SPPF block uses three consecutive 2D convolutions for max-pooling, resulting in faster computation. The C3-PANet architecture combines concatenated feature maps from the SPPF and additional shallow feature maps derived from three distinct levels, ultimately generating a set of aggregated feature maps for prediction purposes. Within CNNs, deeper feature maps possess greater semantic content but less localization information, contrasting with the shallow feature maps. The PANet design employs both up-sampling and down-sampling techniques to establish bottom-up and top-down pathways for the integration of deep and shallow features [51]. In YOLOv5, the C3-PANet translates the extracted feature data into coordinates, categories, and other relevant information.

YOLOv5 head structure generates the final predictions from the feature maps obtained from the backbone and neck through a series of convolutional layers to extract relevant spatial and semantic information. Specifically, the model employs three anchor-based detection heads to conduct dense prediction using aggregated features, with each detection head predicting vectors comprising the coordinates of the estimated bounding box (center, height, width), the confidence score of the prediction, and the classification scores. The head structure contributes significantly to the overall performance of the model.

#### 2.3.2 The loss function

The implemented YOLOv5 model’s total loss comprises confidence loss, classification loss, and bounding box regression loss. The Binary Cross-Entropy (BCE) with Logits loss is utilized for calculating both confidence loss and classification loss, while the Complete Intersection over Union (CIoU) loss is applied for bounding box regression. According to its definition and the obtained training results in the experiments, the classification loss does not contribute to the overall loss in this single-class detection task.

Specifically, the CIoU loss is constructed by incorporating the distance between the centroids of ground truth bounding box B_GT_ and predicted bounding box B, as well as the aspect ratios of B_GT_ and B, as opposed to using the Intersection over Union (IoU) loss:

$$L_{\text{CIoU}}=1-\text{IoU}+\frac{d^{2}\left(\text{b},{\text{b}}^{\text{gt}}\right)}{{\text{c}}^{2}}+\alpha \text{v}$$ (2)

where b and b^gt^ denote the central points of B and B_gt_, d is the Euclidean distance, c is the diagonal length of the smallest enclosing box covering the two boxes, α is a positive trade-off parameter, and v measures the consistency of aspect ratio.

#### 2.3.3 Other pertinent features

The non-maximum suppression (NMS) is integrated to remove the bounding boxes that represent the same object while keeping the most precise one compared to the ground truth in model training. Moreover, the YOLOv5 is equipped with a flexible configuration for adjusting the width and depth of layers, enabling the construction of models with varying complexity to achieve a balance between detection speed and accuracy for diverse applications. Data augmentation techniques are also utilized to increase the variability of the images and improve the generalization capabilities of the trained model.

## 3. Results and discussion

### 3.1 Metrics

Precision, recall, and F1-score are fundamental metrics frequently employed to assess the performance of object detection models. A precision-recall (P-R) curve depicts the relationship between precision (positive predictive value) and recall (sensitivity) across varying confidence scores. Average precision (AP) embodies the balance between precision and recall, and is calculated by estimating the area under the P-R curve to assess the overall performance of object detection models.

$$AP=\int_{0}^{1}p\left(r\right)dr$$ (3)

where *p*(*r*) is the precision at recall value *r*. The Microsoft COCO dataset introduced the 101-point interpolated AP calculation, which is a widely adopted approximation of the area under the P-R curve. This study also utilizes the COCO AP calculation method to evaluate the performance of the implemented models.

From a clinical perspective, precision denotes the ratio of true polyps to all predicted polyps, whereas recall signifies the detection rate of true polyps among all ground truth polyps. Improving model precision reduces unnecessary procedures for healthy individuals, while maintaining high recall allows for the timely identification of patients before lesions progress to cancer.

### 3.2 Model training

The training, validation, and test sets for the experiments are derived from six existing databases: GIANA2017, CVC-ColonDB, GLRC, KUMC, Kvasir-SEG, and CP-CHILD-A. Specifically, 7,276 images from the GIANA2017 database and 200 images from CP-CHILD-A are selected as separate test sets, designated as GIANA2017-T and CP-CHILD-AT, respectively. The remaining images from GIANA2017, CVC-ColonDB, GLRC, and KUMC are divided into the training set GCGK-I (26,657 images) and the validation set GCGK-II (3,966 images). The entire Kvasir-SEG dataset (1,000 images) is incorporated into GCGK-I to create the second-round training set. No images from Kvasir-SEG are included in the validation or test sets. All experiments are carried out in Ubuntu LTS 20.04 with PyTorch 1.12.1, CUDA Toolkit 11.6, and cuDNN 8.3.

**Fig 3:**
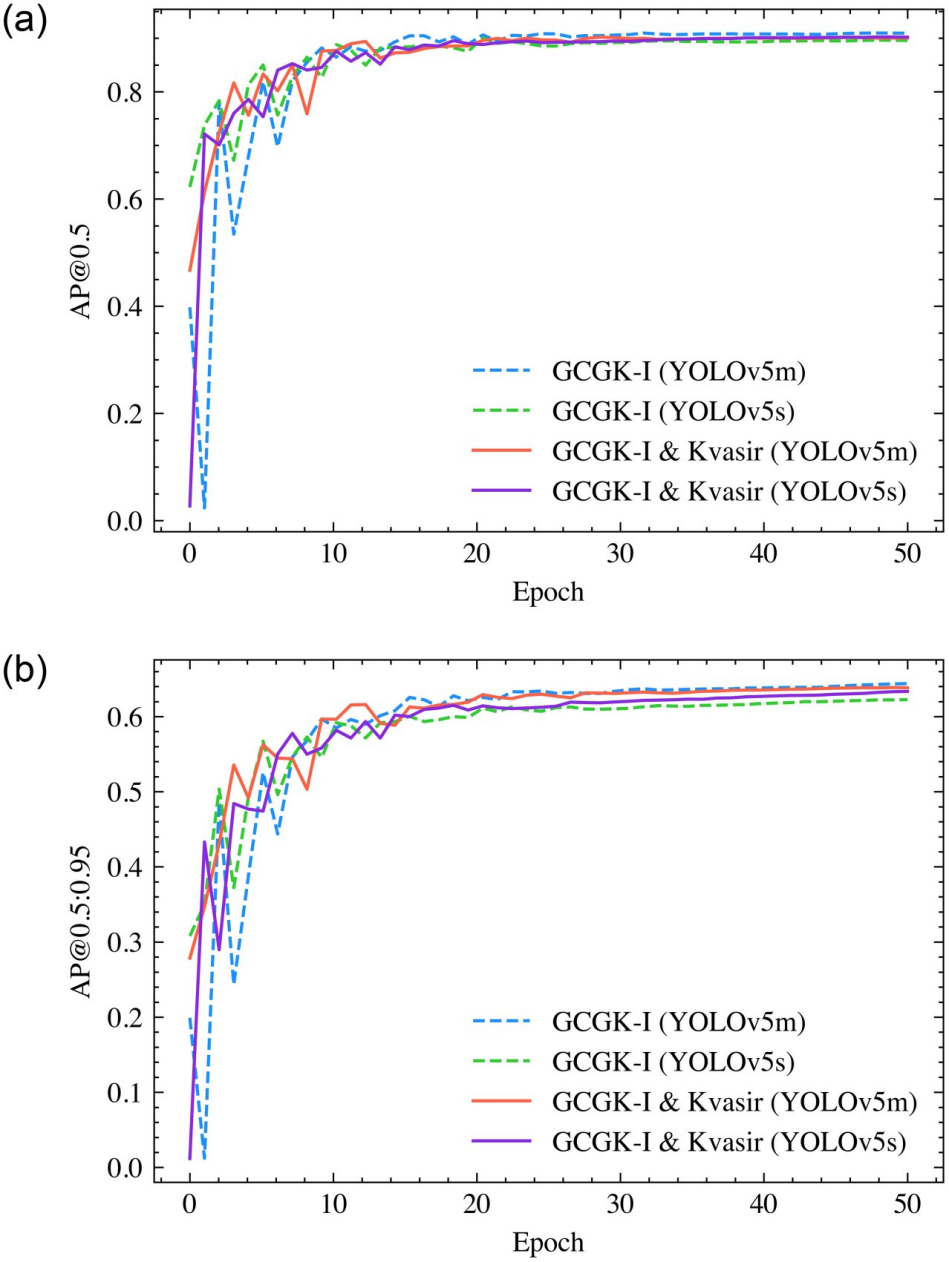
(a) The AP at IoU 0.5 in training process; and (b) the AP at IoU 0.5:0.95 in training process with Adam optimizer.

The image resolution in the training dataset varies and will be resized to 640x640 pixels using the letterbox method. Mosaic augmentation is employed to enhance the model’s generalization capability. The number of epochs is set as 50, batch size as 16, Adam as the optimizer, and learning rate as 0.001. Two distinct YOLOv5 configurations (v5s and v5m) are applied in this study. Fig 3 illustrates the training process incorporating transfer learning. The Average Precision (AP) at Intersection over Union (IoU) of 0.5 is a conventional method for evaluating model performance. The primary challenge metric is the AP at IoU ranging from 0.5 to 0.95 (with increments of 0.05), and the average of these ten APs serving as a single benchmark value for the detection models.

**Fig 4:**
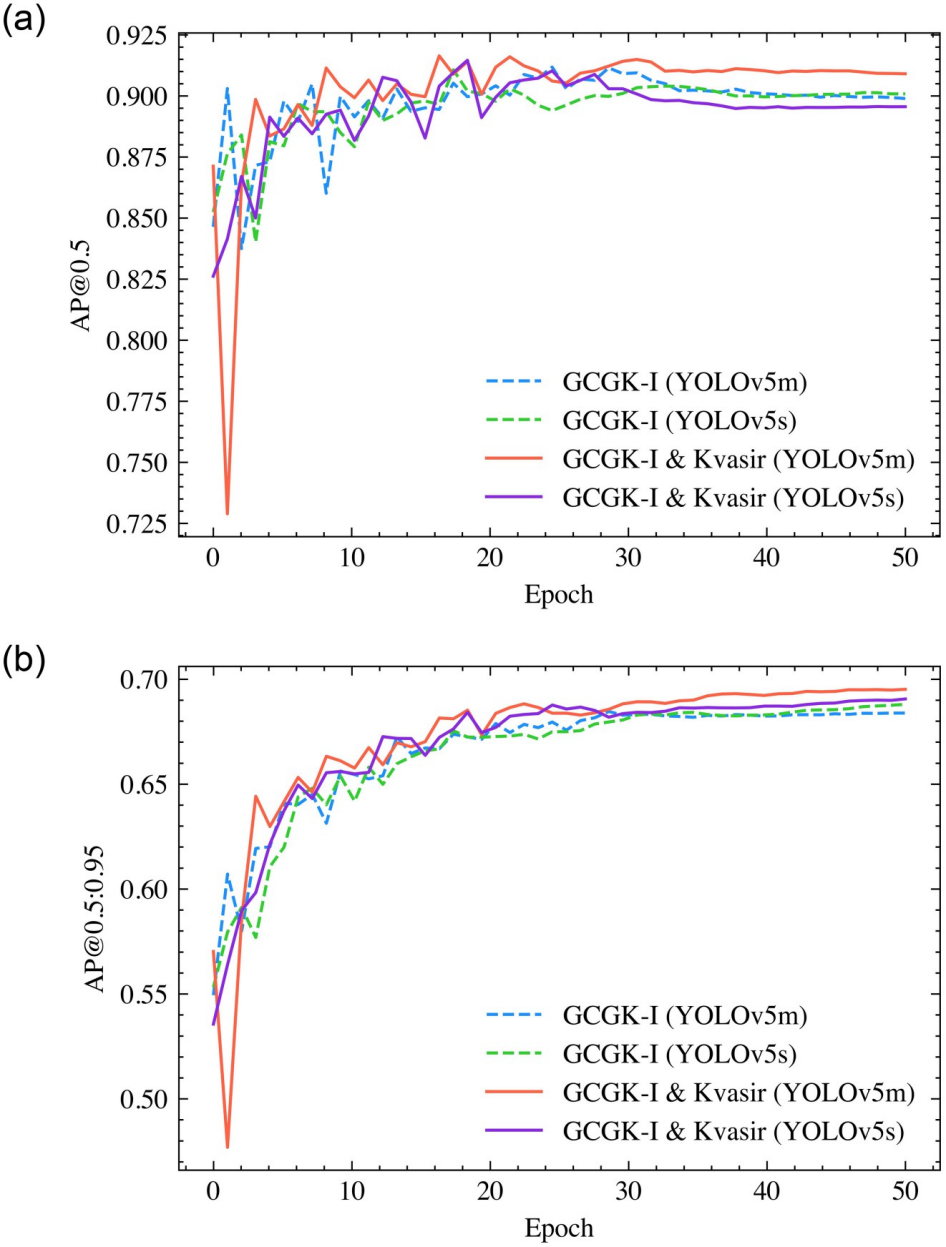
(a) The AP at IoU 0.5 in training process; and (b) the AP at IoU 0.5:0.95 in training process with SGD optimizer.

To examine the influence of the optimizer on training the polyp detection model, Stochastic Gradient Descent (SGD) was employed to substitute Adam, while maintaining other configurations unchanged. Fig 4 presents the training outcomes, demonstrating that the SGD optimizer outperforms Adam in this particular experiment.

### 3.3 Performance validation

**Fig 5:**
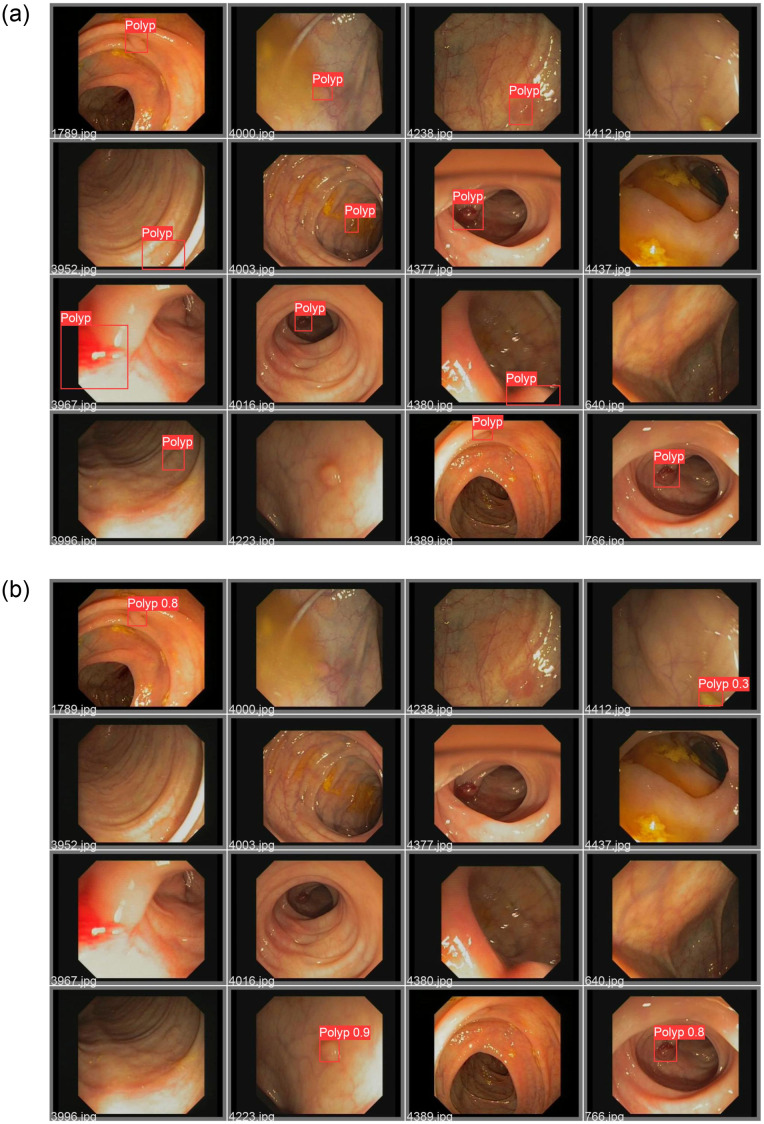
(a) The ground truth of the selected images; and (b) the predicted results of the selected images in GIANA2017-T.

The trained models are tested using GIANA2017-T, with results presented in Tables 2 and 3. It was observed that the trained polyp detection model performed well during testing. The training set, composed of GCGK-I and Kvasir-SEG, utilized the YOLOv5m model with an SGD optimizer, yielding the best results in the experiment. The expansion of the training dataset indeed impacted the result significantly, while the model complexity seems not to be a substantial factor in the test. Alterations in depth and width configurations demonstrated noticeable differences in object detection performance for the Microsoft COCO dataset and other object detection datasets, though not in this particular test. A possible explanation for this is the relatively small and simple nature of the polyp database, which constrains the development of more accurate and robust polyp detection models. Fig 5 displays the ground truth and predicted results of several selected images from GIANA2017-T.

**Table 2:** Results of model trained with Adam optimizer on test set GIANA2017-T.

Training Set	Model	Precision	Recall	AP@0.5	AP@0.5:0.95
**GCGK-I**	YOLOv5m	0.81	0.656	0.742	0.4
**GCGK-I**	YOLOv5s	0.819	0.667	0.744	0.399
**GCGK-I & Kvasir**	YOLOv5m	0.793	0.653	0.73	0.416
**GCGK-I & Kvasir**	YOLOv5s	0.812	0.665	0.747	0.414

**Table 3:** Results of model trained with SGD optimizer on test set GIANA2017-T.

Training Set	Model	Precision	Recall	AP@0.5	AP@0.5:0.95
**GCGK-I**	YOLOv5m	0.867	0.674	0.768	0.401
**GCGK-I**	YOLOv5s	0.806	0.691	0.751	0.404
**GCGK-I & Kvasir**	YOLOv5m	0.9	0.717	0.811	0.467
**GCGK-I & Kvasir**	YOLOv5s	0.886	0.718	0.807	0.462

Besides, the state-of-the-art object detection model Faster R-CNN and RetinaNet are also implemented to compare with YOLOv5 on the test set. Faster R-CNN, a two-stage object detection approach, employs a Region Proposal Network (RPN) to generate potential bounding boxes and a classifier to refine and classify these proposals. RetinaNet introduces the Focal Loss function to address foreground-background class imbalance issue in single-stage detectors. The analysis reveals that YOLOv5 achieves superior performance compared to Faster R-CNN and RetinaNet, as shown in Table 4.

**Table 4:** Comparison of models on test set GIANA2017-T.

Training Set	Model	AP@0.5	AP@0.75	AP@0.5:0.95
**GCGK-I & Kvasir**	Faster R-CNN	0.769	0.454	0.443
**GCGK-I & Kvasir**	RetinaNet	0.651	0.311	0.345
**GCGK-I & Kvasir**	YOLOv5m	0.811	0.446	0.467

Particular emphasis is placed on false negative predictions, as failure to detect polyps can lead to serious consequences for patients. Upon examining the test results, several representative cases of lesions undetected by the implemented YOLOv5 model are presented in Table 5. False negatives were observed in the following scenarios: small and flat polyps without superficial congestion, polyps obscured by feces or foam, polyps with a color similar to the adjacent mucosa, polyps situated near the intestinal wall folds, and poorly illuminated captured images.

**Table 5:** The list of some representative predictions in the test set.

Image ID	Ground Truth	Prediction	Possible Reasons
**3996, 4003, 4233, 4238**	Polyp	FN ^a^	Polyps are flat in shape, small in diameter, colors are similar to the surrounding mucosa.
**3952, 4389**	Polyp	FN	Polyps located nearby or even covered by intestinal folds, colors are similar to the surrounding mucosa.
**4380, 3967**	Polyp	FN	The images only capture partial polyps, and cannot fully show the full shape of polyps.
**4412**	None	FP ^b^	Misjudged feces as polyps.
**4377, 4016**	Polyp	FN	Polyps are far away from the lens due to the shooting angle, or the light is insufficient.
**4000**	Polyp	FN	Part of the polyp is covered by fecal water, polyps are smaller, color similar to the surrounding mucosa.
**1789, 766**	Polyp	TN ^c^	Accurate predictions.
**4437, 640**	None	TP ^d^	Accurate predictions.

^a^ FN–False Negative,

^b^ FP–False Positive,

^c^ TN–True Negative,

^d^ TP–Ture Positive.

In addition to the instances of misdiagnosis and missed diagnosis demonstrated in the experiments, it is likely that the trained model would predict false positives in these situations based on clinical experience:

1. Submucosal bulging lesions protruding from the intestine such as lipomas, mesenchymal tumors, fibromas, etc. The surface mucosa of this lesion is normal, and the actual lesion is located in the submucosa, which can be easily mistaken as polyps.
2. Inadequate intestinal preparation in some patients may result in undigested food residues adhering to the intestinal lumen’s surface, leading the model to misdiagnose these residues as colonic polyps.
3. When the self-purification capability of the colonoscopy decreases or the endoscopist fails to rinse the lens properly, the lens would be blurry and has water droplets. The images captured will be blurred and distorted, and unable to accurately represent the presence of colonic polyps.
4. Nipple hypertrophy in the anus may also appear as a protrusion from the intestinal lumen in the object detection model’s perspective.
5. Colonoscopy often needs to go into the end of the ileum to observe, and the lymphoid follicles present at the end of the ileum are similar to certain small colonic polyps.
6. Varicose veins in the rectum, such as earthworm-like or beaded bulges.
7. The colonic diverticulum manifests as an irregular indentation within the intestinal cavity, forming a distinct contrast in color with the surrounding intestinal lumen, which can be misidentified as polyps.

Juvenile polyps are the most prevalent pathological subtype found in children who suffering from this condition, although adenomatous, proliferative, and inflammatory polyps are also observed. Clinical symptoms of colon polyps in children include hematochezia, abdominal pain, constipation, anemia, and occasionally, intestinal obstruction. Typically, pediatric polyps are spherical or hemispherical, pedunculated, and exhibit villous or lobulated surfaces; some may be piebald. Wang et al. developed a classification network using colonoscopy images gathered from the Gastrointestinal Endoscopy Unit of Hunan Children’s Hospital. The original dataset did not include bounding box annotations. Consequently, an experienced endoscopist selected and labeled 200 images from the dataset were to create the test set CP-CHILD-AT utilized in this study. The objective of this experiment is to investigate the necessity for age-specific colonoscopy polyp datasets. Table 6 presents the performance of the proposed models in detecting polyps within the CP-CHILD-AT test set.

**Table 6:** Comparison of models on test set CP-CHILD-AT.

Training Set	Model	AP@0.5	AP@0.75	AP@0.5:0.95
**GCGK-I & Kvasir**	Faster R-CNN	0.922	0.785	0.678
**GCGK-I & Kvasir**	RetinaNet	0.918	0.782	0.685
**GCGK-I & Kvasir**	YOLOv5m	0.935	0.871	0.755

**Fig 6:**
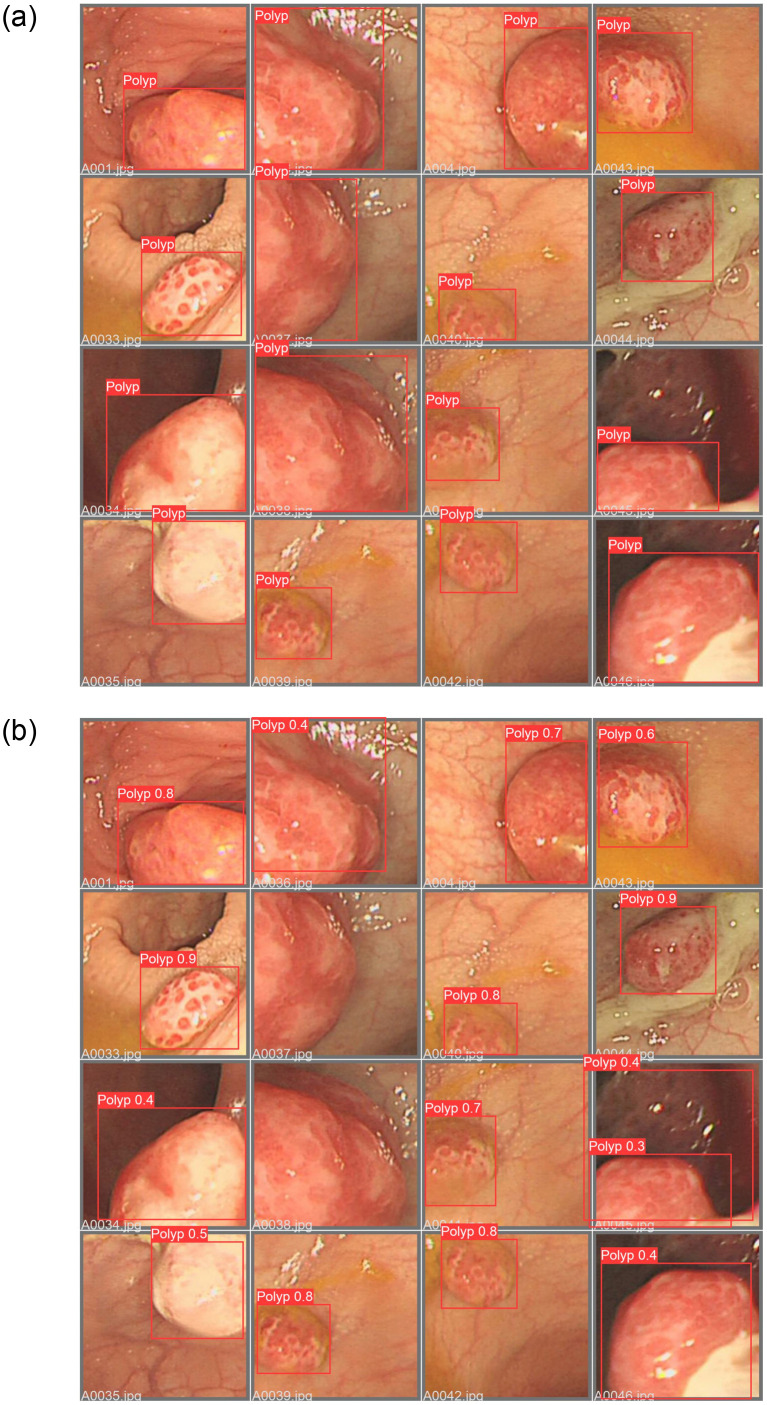
(a) The ground truth of the images; and (b) the predicted results of the images in CP-CHILD-AT.

The results indicate that the model trained on the GCGK-I and Kvasir datasets demonstrates robust performance on the test set. Considering factors such as polyp morphology and generalization, constructing dedicated datasets for adult or pediatric colonoscopy images for polyp detection is not advised. Instead, training with high-quality mixed datasets can be considered. Fig 6 presents the ground truth and predicted results for selected images from the CP-CHILD-AT dataset with the implemented model.

The experimental findings in this study underscore the substantial influence that different datasets have on the outcomes of polyp detection when employing contemporary object detection models. Consequently, the current limited datasets might not adequately demonstrate the full capability or limitations of previous proposed detection models. This insight indicates a prospective shift in research priorities, suggesting that the creation of a more comprehensive and diverse dataset for polyp detection might substantially augment progress in this field, rather than focusing solely on the development of novel models. This strategic transition could potentially overcome the current limitations of these object detection models in both research and clinical practice.

### 3.4 Qualify management framework in polyp detection

The currently available polyp datasets are inadequate, and accessing existing databases necessitates strict oversight by the database owner. As a result, investing significant effort in reconstructing CNN networks and evaluating polyp detection model performance has limited value due to the constraints of these datasets. The colonoscopy image datasets primarily consist of images containing polyps, with each pixel in a dataset image considered a training signal. In fact, numerous pixels with negative signals (background) exist in each image, and implementing a loss function that emphasizes positive signals (polyp pixels), such as focal loss, could enhance detection performance [52]. The MICCAI2017 dataset contains 38 videos, and the SUN Colonoscopy Video Database comprises 49,136 polyp frames extracted from 100 distinct polyps of 99 registered patients. Segmenting these videos by frame could yield tens of thousands of images for use as training data, while the extracted images actually hold minimal information, as most endoscopic video sequences primarily feature a few specific polyps viewed from various angles. To a certain extent, the transfer learning strategy can mitigate this problem. However, if the database remains limited and unimproved, the effect of such enhancement will be marginal.

**Fig 7:**
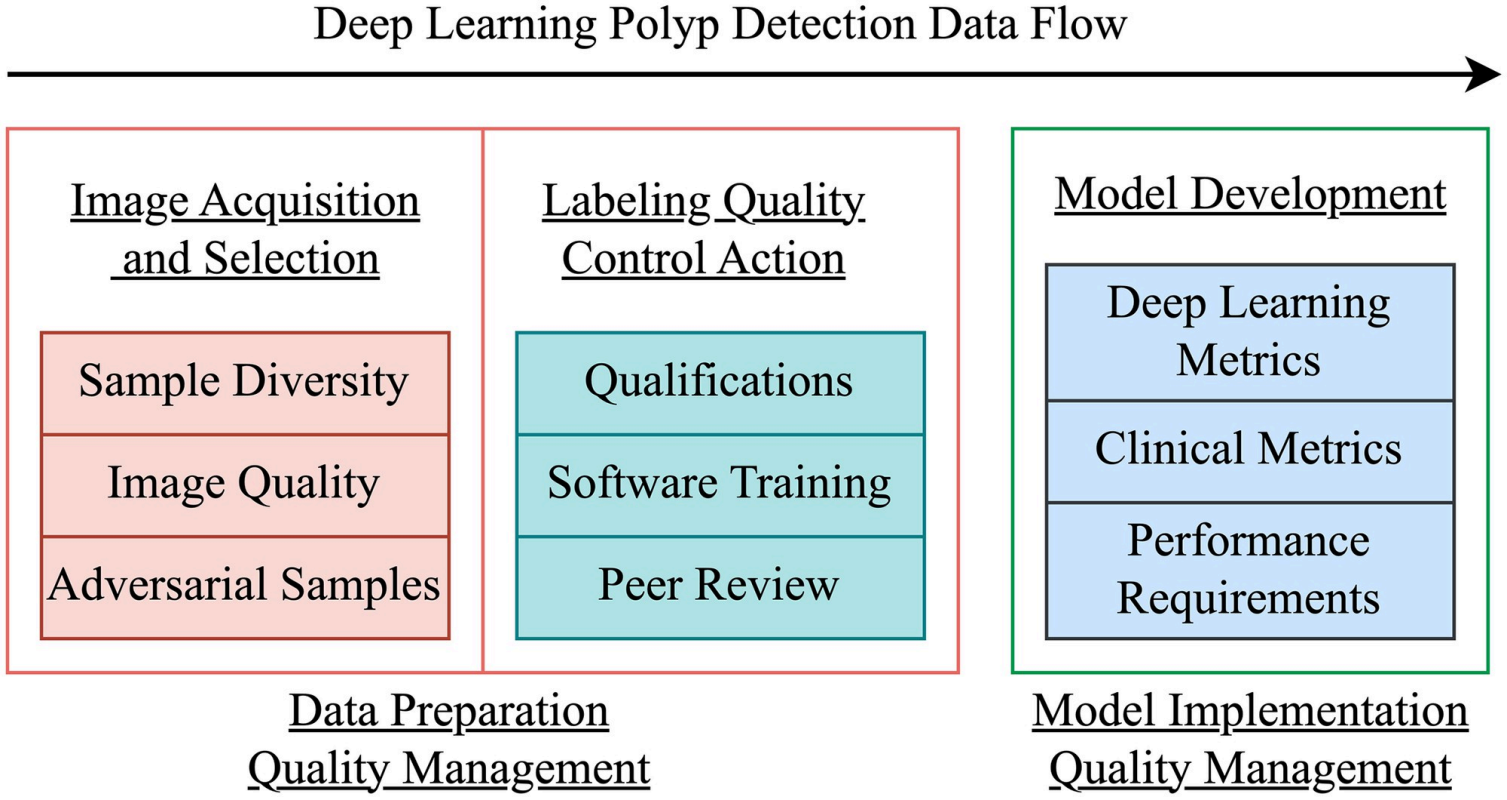
The quality management and control framework in model development.

The systematic quality management framework to ensure deep learning polyp detection performance is recommended in future applications, as presented in Fig 7. The experiment indicates that dataset construction significantly influences detection accuracy. The first component of the framework is to adopt guidelines to ensure the dataset preparation quality through the emphasis on sample diversity, image quality, and adversarial samples. Labeling quality is another critical factor in dataset preparation, as labeled results may deviate from actual values due to the labeler’s training, experience, and subjectivity, similar to colonoscopy procedures. Therefore, the second component of the framework proposes specifying labeler qualification requirements and conducting a comprehensive study of the annotation software for a predetermined duration. The annotated images must be peer-reviewed before incorporated into the dataset. Presently, implemented polyp detection models are predominantly assessed using conventional deep learning performance metrics, which are intimately connected to disease diagnosis. The framework further recommends employing both deep learning and clinical metrics to evaluate the detection model and establish specific performance criteria as the third component.

The dataset preparation and model implementation quality management constitute a quality control framework for deep learning-based colorectal polyp detection. The proposed framework addresses existing challenges in deep learning polyp detection and enhances dataset quality, paving the way for the creation of a renowned public polyp image dataset. More importantly, after clarifying the specific indicators and performance requirements of the polyp detection model, the specific neural network models that meet the clinical needs can be designed intentionally, rather than merely adapting object detection models from the field computer vision.

## 4. Conclusion

This paper investigates the current constraints in the construction of clinical polyp detection models using deep learning techniques. A one-stage object detection model based on YOLOv5 was implemented for colorectal polyp detection, achieving satisfactory accuracy in the test dataset through the strategic application of transfer learning. The diversity of the training set was identified as a crucial factor constraining deep learning performance in current polyp detection after evaluating the model across various training and test datasets. Consequently, the creation of diverse and high-quality polyp image datasets is of utmost importance. The proposed models need to be assessed from both computer vision and clinical perspectives to enhance their performance. In addition, a polyp detection quality management framework is recommended to reduce errors in data preparation and establish appropriate performance metrics for model development. Future research intends to collect and annotate more diverse data, including polyps of differing sizes, shapes, stages, and imaging conditions, in order to construct a more comprehensive dataset. Subsequent initiatives will concentrate on developing specifical models for polyp detection to effectively learn the unique characteristics of polyps and gastrointestinal images. This advancement could assist endoscopists in detecting polyps more accurately and efficiently, and contributing to the evolution of a sustainable healthcare system.
